# Antimicrobial Resistance and Genotypic Diversity of *Campylobacter* Isolated from Pigs, Dairy, and Beef Cattle in Tanzania

**DOI:** 10.3389/fmicb.2015.01240

**Published:** 2015-11-12

**Authors:** Isaac P. Kashoma, Issmat I. Kassem, Anand Kumar, Beda M. Kessy, Wondwossen Gebreyes, Rudovick R. Kazwala, Gireesh Rajashekara

**Affiliations:** ^1^Food Animal Health Research Program, Ohio Agricultural Research and Development Center, The Ohio State UniversityWooster, OH, USA; ^2^VPH-Biotec Global Consortium; ^3^Faculty of Veterinary Medicine, Sokoine University of AgricultureMorogoro, Tanzania; ^4^Department of Veterinary Preventive Medicine, College of Veterinary Medicine, The Ohio State UniversityColumbus, OH, USA

**Keywords:** *Campylobacter*, MLST, food animals, food safety, antimicrobial resistance

## Abstract

Foodborne *Campylobacter* infections pose a serious threat to public health worldwide. However, the occurrence and characteristics of *Campylobacter* in food animals and products remain largely unknown in Tanzania. The objective of this study was to determine the prevalence, antibiotic resistance, and genetic profiles (sequence types, STs) of *Campylobacter* isolated from feces of pigs and dairy and beef cattle in Tanzania. Overall, 259 (~30%) of 864 samples were positive for *Campylobacter* spp, which were detected in 32.5, 35.4, and 19.6% of the pig, dairy, and beef cattle samples, respectively. Multiplex PCR analysis identified 64.5 and 29.3% of the *Campylobacter* isolates as *C. coli* and *C. jejuni*, respectively. The majority (91.9%) of the isolates from pig samples were identified as *C. coli*, while *C. jejuni* accounted for 65.5% of the isolates from cattle. Antimicrobial susceptibility testing using the disk diffusion assay and the broth microdilution method revealed resistance to: ampicillin (Amp) (70.3% and 75.7%, respectively), gentamicin (Gen) (1.8% and 12.6%), streptomycin (Str) (65.8 and 74.8%), erythromycin (Ery) (41.4 and 48.7%), tetracycline (Tet) (18.9 and 23.4%), and ciprofloxacin (Cip) (14.4 and 7.2%). Resistance to nalidixic acid (Nal) (39.6%), azithromycin (Azm) (13.5%), and chloramphenicol (Chl) (4.5%) was determined using the disk diffusion assay only, while resistance to tylosin (Tyl) (38.7%) was quantified using the broth microdilution method. Multilocus sequence typing of 111 *Campylobacter* isolates resulted in the identification of 48 STs (26 *C. jejuni* and 22 *C. coli*) of which seven were novel (six *C. jejuni* and one *C. coli*). Taken together, this study revealed the high prevalence, genetic diversity and antimicrobial resistance of *Campylobacter* in important food animals in Tanzania, which highlights the urgent need for the surveillance and control of *Campylobacter* in this country.

## Introduction

*Campylobacter* spp. are among the most common etiological agents of foodborne bacterial gastroenteritis in humans worldwide, accounting for an estimated 500 million infections per year globally (Ruiz-Palacios, 2007; WHO, 2013). The number of reported cases of campylobacteriosis is high in developed countries (Scallan et al., 2011; EFSA and ECDC, 2014), while the disease remains under reported in developing countries due to the absence of regular surveillance programs (Coker et al., 2002). In Tanzania, *Campylobacter* has been reported to affect up to 20% of children under 5 years old (Jacob et al., 2011; Deogratias et al., 2014). *Campylobacter* is increasingly becoming a major problem in Sub-Sahara Africa where the number of infections is predicted to double by the year 2020 (Coker et al., 2002). Furthermore, deficiencies in food safety regulations and limited epidemiological data in many African countries, including Tanzania, hamper the assessment, surveillance, and control of *Campylobacte*r infections. Therefore, in these countries, studies that address the occurrence and antimicrobial resistance of *Campylobacter* in food animals are of paramount importance.

*Campylobacter* is zoonotic and various food animals including poultry, pigs, and cattle are implicated as important reservoirs (Man, 2011; Sahin et al., 2015). Recent studies have shown that the contributions of non-poultry associated *Campylobacter* to human infections are considerable and warrant investigation (Ragimbeau et al., 2008; Wilson et al., 2008). For example, it was shown that cases of human *Campylobacter* infections can be attributed equally to cattle and poultry sources in certain countries (Wilson et al., 2008; de Haan et al., 2010). Studies from different countries reported that *Campylobacter* prevalence can range from 12 to 100% in dairy herds (Gilpin et al., 2008; Ragimbeau et al., 2008; Pradhan et al., 2009; Sanad et al., 2011), 5.4 to 83% in beef cattle (Salihu et al., 2009; Haruna et al., 2013), and 46 to 100% in pigs (Saenz et al., 2000; Pezzotti et al., 2003; Payot et al., 2004; Litrup et al., 2007; Mdegela et al., 2011). While *Campylobacter jejuni* can constitute the predominant species isolated from cattle (Ragimbeau et al., 2008; Sanad et al., 2011), *Campylobacter coli* is more frequently isolated from pigs (Saenz et al., 2000; Mdegela et al., 2011). In addition to potential contamination of milk and carcasses at slaughter, *Campylobacter* colonization of cattle and pigs pose a serious risk for contamination of surface and sub-surface water during disposal of abattoir effluents and animal slurries. This might further contribute to the transmission of *Campylobacter* to other food animals or directly to humans (Minihan et al., 2004; Devane et al., 2005; Garrett et al., 2007). Consequently, defining the role of cattle and pigs as reservoirs for these pathogens might be important for understanding the epidemiology of *Campylobacter* in Tanzania.

Most *Campylobacter* infections in humans are self-limiting and do not require antimicrobial therapy. However, in systemic infections or in immunocompromised individuals, erythromycin (Ery) and fluoroquinolones are used as the drugs of choice. However, studies have reported an increase in the resistance of *Campylobacter* to various antimicrobials, including the drugs of choice (Van Looveren et al., 2001; Chen et al., 2010; Cody et al., 2010; Rozynek et al., 2010). The rise in antimicrobial resistant *Campylobacter* has been linked to the use of antimicrobials in veterinary medicine and in farming practices (White et al., 2002; Zhu et al., 2006). While antimicrobial resistant *Campylobacter* has been reported worldwide, the situation might be more severe in developing countries where there is widespread and largely uncontrolled use of antimicrobials (Byarugaba, 2004; Kariuki, 2010). This is particularly important, because some of the resistant isolates have been suspected to spread from food animals to humans (Rozynek et al., 2010). Therefore, analysis of antimicrobial resistance of *Campylobacter* isolated from food animals in developing countries is needed to better manage cognate infections and mitigate emergence of antimicrobial resistant strains. Subsequently, in this study, we investigated the prevalence, antimicrobial resistance, and genetic diversity of *Campylobacter* isolated from pigs, dairy, and beef cattle from three different geographical regions in Tanzania.

## Materials and methods

### Geographical locations and sample collection

A cross-sectional sampling was conducted between April 2013 and March 2014. Samples were collected from pigs, dairy, and beef cattle from three geographically distinct regions of Tanzania, namely Arusha in Northern Tanzania: Iringa in Southwestern Tanzania, and Morogoro in Eastern Tanzania. These locations were strategically selected, because they are among the regions that hold the largest populations of farmed animals in Tanzania. A total of 864 samples: pig feces (*n* = 458), beef cattle feces (*n* = 214), and dairy cattle feces (*n* = 192) were collected from the three regions [Arusha (*n* = 189; 82 pig, 47 dairy, and 60 beef samples), Iringa (*n* = 150; 66 pig, 32 dairy, and 52 beef samples), and Morogoro (*n* = 525; 310 pig, 113 dairy, and 102 beef samples)]. Beef cattle feces samples (10 g) were randomly and aseptically collected from the colon during slaughter at the abattoirs. Similarly, 10 g of fresh pen-floor fecal samples were aseptically collected from dairy cattle and pigs on farms. Samples were placed on ice and immediately shipped to the laboratory for *Campylobacter* isolation. All samples were processed within 24–48 h after collection.

### Isolation of *Campylobacter* from fecal samples

*Campylobacter* was isolated as described previously (Kashoma et al., 2014, 2015). Approximately 2 g of feces were suspended in 9 mL of maximum recovery diluent (MRD) (Neogen, USA). One milliliters of the suspension was added to 9 mL of Preston broth containing *Campylobacter* growth and selective supplements (SR0117E and SR0232; Oxoid, England). The suspensions were then incubated at 42°C for 48 h in airtight jars containing the Campy Pouch system (Becton Dickinson and Co., Maryland, USA) to generate microaerobic conditions. After incubation, 100 μL of the suspension was spread onto a modified charcoal cefoperazone deoxycholate agar (mCCDA) plate (Oxoid) containing a *Campylobacter* selective supplement (SR0155E, Oxoid) and incubated for 48 h at 42°C under microaerobic conditions. Three to five colonies suspected as *Campylobacter* were selected from each mCCDA plate and further purified using Muller-Hinton (MH; Difco, MD) agar plates containing a *Campylobacter* selective supplement (SR0117E, Oxoid). Pure cultures were stored at −80°C in MH broth supplemented with 30% glycerol (vol/vol) until further analysis.

### Identification of *Campylobacter* species using PCR

For PCR analysis, bacterial DNA lysates were prepared from fresh pure *Campylobacter* cultures using the boiling method as previously described (Kashoma et al., 2014, 2015). In cases where no PCR products were detected, template DNA was prepared using QIAamp DNA Mini Kit (QIAGEN, Hilden, Germany) according to the manufacturer's instructions. Confirmation and speciation of putative *Campylobacter* was performed by multiplex-PCR (mPCR) as described previously (Linton et al., 1997; Denis et al., 1999; Yamazaki-Matsune et al., 2007). Isolates that were positive for the genus-specific PCR but negative for the *C. coli* and *C. jejuni*-specific PCR were designated as other thermophilic campylobacters (OTC). *C. jejuni* 81–176 (wild-type strain) and *C. coli* (ATCC 33559) were used as positive controls, while standard-grade laboratory water was used as a no template (negative) control.

### Antimicrobial susceptibility analysis

Antimicrobial resistance analysis was performed on 111 *Campylobacter* isolates (42 *C. jejuni* and 69 *C. coli*) that were randomly selected to represent different animal hosts and geographical locations. The antimicrobials tested are representatives of the drugs used for humans and in the animal industry in Tanzania (Kashoma et al., 2015; Komba et al., 2015). The analysis was conducted using the Kirby-Bauer disk diffusion and the broth microdilution methods as described previously (Luber et al., 2003; Lehtopolku et al., 2012; Kashoma et al., 2015). Both tests were performed in accordance to the recommendations of the Clinical Laboratory Standards Institute (CLSI, 2012). The results were interpreted as susceptible, intermediately resistant, or resistant according to the CLSI (2012) or the ROSCO MIC for veterinary isolates (ROSCO, 2007) guidelines (Kashoma et al., 2015). Multi-drug Resistance (MDR) was defined as resistance to three or more antimicrobial agents (Hakanen et al., 2003).

In the Kirby-Bauer disk diffusion test, nine antimicrobial agents (Oxoid, UK) were tested at the following concentrations: 10 μg ampicillin (Amp), 5 μg ciprofloxacin (Cip), 15 μg Ery, 30 μg nalidixic acid (Nal), 10 μg streptomycin (Str), 30 μg tetracycline (Tet), 15 μg azithromycin (Azm), 10 μg gentamicin (Gen), and 30 μg chloramphenicol (Chl). The antimicrobial discs were placed on the surface of culture plates and the diameter of the zone of inhibition was measured after 24 h of microaerobic incubation at 42°C.

For the broth microdilution test and the determination of the minimum inhibitory concentration (MIC), 96-well plates containing two-fold serial dilutions of the antimicrobial agents were used as described previously (Ge et al., 2013; Kashoma et al., 2015). The antimicrobial agents tested included Amp, Cip, Ery, Gen, tylosin (Tyl), Str, and Tet. MIC values were defined as the lowest concentration of an antimicrobial agent that produced no visible growth and the results were confirmed spectrophotometrically using a microplate reader (Multiskan® Spectrum, Thermo Scientific, USA) (Ge et al., 2013). In both assays, *C. jejuni* 81–176 and *C. coli* (ATCC 33559) were used as positive control strains.

### Multilocus sequence typing (MLST) analysis

In order to determine the genetic diversity of the *Campylobacter* isolates and their relationship to existing clonal complexes (CC) and sequence types (STs), 111 isolates (42 *C. jejuni* and 69 *C. coli*) which were also tested for antimicrobial resistance were analyzed by MLST as described previously (Dingle et al., 2001; Sanad et al., 2011). Briefly, loci from seven housekeeping genes (*aspA, glnA, gltA, glyA, pgm, tkt, and uncA*) were amplified using PCR and specific primers (Dingle et al., 2001). The size of the amplicons was confirmed using agarose gel electrophoresis. PCR products were then treated with ExoSAP (Affymetrix Inc., USA), sequenced in both directions. The forward and reverse sequences were aligned using ClustalW (www.ebi.ac.uk/clustalw), allele profiles were determined by BLAST analysis using the single-locus query function, while STs were assigned using the allele profile query function available in the MLST *Campylobacter* database (http://pubmlst.org/campylobacter). STs were then traced to their respective CC using BURST (http://pubmlst.org/).

### Statistical analysis

The prevalence and antimicrobial resistance of *Campylobacter* from pigs, dairy, and beef cattle in all three regions were compared using the Chi-squared (χ^2^) test. A value of *P* < 0.05 was considered statistically significant. Agreement between the two antimicrobial resistance tests was determined using the Kappa statistic (Luber et al., 2003). A Kappa value of 100% indicates total agreement between the classifiers.

## Results

### The prevalence of *Campylobacter* in feces sampled from cattle and pigs

*Campylobacter* were detected in 259 (30%) of 864 fecal samples and were distributed as follows: a total of 149 (32.5%), 68 (35.4%), and 42 (19.6%) isolates were retrieved from pig, dairy, and beef cattle fecal samples, respectively. *C. jejuni* and *C. coli* constituted the majority of the isolates and were detected in 76 (8.8%) and 166 (19.3%) of the samples, respectively (Table 1). There was no significant difference (*P*>0.05) in the total number of *C. jejuni* isolated from fecal samples obtained from either dairy (21.9%) or the beef cattle (14.0%). Significantly (*P* < 0.0001) more *C. coli* were isolated from pigs (29.9%) in comparison to cattle (7.4%), while *C. jejuni* was significantly higher (*P* < 0.0001) in cattle (17.7%) than in pig (0.9%) samples. Furthermore, the occurrence of *Campylobacter* in animals varied according to geographic location of sampling sites. Specifically, *Campylobacter* were retrieved from 34.1% of all fecal samples collected from Morogoro, which was significantly higher (*P* < 0.05) than those from Arusha (27.5%) and Iringa (18.7%), respectively (Table 1). Moreover, total *C. coli* prevalence was significantly higher (*P* < 0.05) in Morogoro (5.9%) than in Arusha (1.7%) or Iringa (1.9%). The combined prevalence of *C. jejuni* in beef and dairy cattle feces from Morogoro (23.2%; 50 *C. jejuni* retrieved from 215 fecal samples) was significantly higher (*P* < 0.05) in comparison to that from Arusha (12.2%) and Iringa (10.7%), respectively. However, no significant difference (*P*>0.05) in the prevalence of *C. jejuni* in beef and dairy cattle feces was observed between Arusha and Iringa.

**Table 1:** **The prevalence of *Campylobacter* species in pig, dairy, and beef cattle feces collected from three geographical locations in Tanzania**.

**Region**	**Sources**	**Prevalence (%); positive samples/No. of samples tested**	**Positive samples No. %**			
			***C. jejuni***	***C. coli***	***C. jejuni/C. coli*^a^**	**OTC^b^**
Arusha	Pig	36.6% (30/82)	2 (2.4%)	25 (30.5%)	1 (1.2%)	2 (2.4%)
	Dairy	29.8% (14/47)	7 (14.9%)	6 (12.8%)	–	1 (2.1%)
	Beef	13.3% (8/60)	6 (10.0%)	1 (1.7%)	–	1 (1.7%)
Iringa	Pig	22.7% (15/66)	–	13 (19.7%)	1 (1.5%)	1 (1.5%)
	Dairy	25.0% (8/32)	5 (15.6%)	2 (6.3%)	–	1 (3.1%)
	Beef	9.6% (5/52)	4 (7.7%)	1 (1.9%)	–	–
Morogoro	Pig	33.6% (104/310)	2 (0.7%)	99 (31.9%)	1 (0.3%)	2 (0.7%)
	Dairy	40.7% (46/113)	30 (26.6%)	14 (12.4%)	1 (0.9%)	1 (0.9%)
	Beef	27.5% (29/102)	20 (19.6%)	6 (5.9%)	1 (1.0%)	2 (1.9%)
Total		29.98% (259/864)	76 (8.8%)	167 (19.2%)	5 (0.6%)	11 (1.3%)

a Isolates that were positive for both C. jejuni and C. coli.

b OTC, other thermophilic Campylobacter species.

### Antimicrobial susceptibility of the *C. jejuni* and *C. coli* isolates

Analysis of the Kirby-Bauer disk diffusion assay showed that 106 of the 111 isolates (95.5%) were resistant to one or more antimicrobial agents, whereas five (4.5%) isolates were pan-susceptible to all antimicrobials tested (Table 2). Twenty-one isolates (18.9%; six *C. jejuni* and 15 *C. coli*) were resistant to a single antimicrobial agent and 22 isolates (19.8%; 10 *C. jejuni* and 12 *C. coli*) showed resistance to two antimicrobial agents (Table 2). Sixty-three (56.8%) of all isolates (24 *C. jejuni* and 39 *C. coli*) were classified as MDR. Of the MDR isolates, 38 (60.3%) were from pig, 15 (23.8%) from dairy, and 10 (15.9%) from beef cattle feces. Two *C. jejuni* isolates recovered from dairy cattle were resistant to Gen, whereas 9.0% of all isolates (four *C. jejuni* and six *C. coli*) were resistant to Chl (Table 2). Approximately 14.4% (six *C. jejuni* and 10 *C. coli*) and 13.5% (six *C. jejuni* and nine *C. coli*) of the isolates were resistant to Cip and Azm, respectively (Tables 2, **4**). Twenty-one (18.9%) isolates were shown to be resistant to Tet, 46 (41.4%) to Ery, and 44 (39.6%) to Nal. In addition, 65.8% (34 *C. jejuni* and 39 *C. coli*) and 70.3% (28 *C. jejuni* and 50 *C. coli*) of isolates were resistant to Str and Amp, respectively (Tables 2, **4**). While resistance to Str was significantly higher (*P* < 0.05) in isolates recovered from cattle in comparison to those from pigs, there were no significant differences (*P*>0.05) in resistance associated with the remaining antimicrobials.

**Table 2:** **Antimicrobial resistance of *C. jejuni* and *C. coli* isolated from pig, dairy, and beef cattle samples**.

**Resistance profile^a^**	**Resistant isolates**			**Source of isolate and number of resistant isolates (%)**					
				**Pig**		**Dairy cattle**		**Beef cattle**	
	**No. (%)**	***C. jejuni* (*n* = 42)**	***C. coli* (*n* = 69)**	***C. jejuni* (*n* = 2)**	***C. coli* (*n* = 67)**	***C. jejuni* (*n* = 26)**	***C. coli* (*n* = 1)**	***C. jejuni* (*n* = 14)**	***C. coli* (*n* = 1)**
Pan-susceptible	5 (4.5)	2(4.8)	3(4.4)	0	3 (4.5)	2 (7.7)	0	0	0
Amp	12 (10.8)	2(4.8)	10(14.5)	0	10 (14.9)	1 (3.9)	0	1 (7.1)	0
Ery	2 (1.8)	0	2(2.9)	0	2 (3.0)	0	0	0	0
Str	4 (3.6)	1(2.4)	3(4.4)	0	3 (4.5)	0	0	1 (7.1)	0
Nal	3 (2.7)	3(7.2)	0	0	0	2 (7.7)	0	1 (7.1)	0
Amp/Cip	1 (0.9)	0	1(1.5)	0	1 (1.5)	0	0	0	0
Str/Tet	3 (2.7)	1(2.4)	2(2.9)	0	2 (3.0)	1 (3.9)	0	0	0
Azm/Ery	2 (1.8)	0	2(2.9)	0	2 (3.0)	0	0	0	0
Amp/Str	11 (9.9)	7(16.7)	4(5.8)	1 (50.0)	4 (6.0)	4 (15.4)	0	2 (14.3)	0
Nal/Str	2 (1.8)	1(2.4)	1(1.5)	0	1 (1.5)	1 (3.9)	0	0	0
Amp/Ery	3 (2.7)	1(2.4)	2(2.9)	0	2 (3.0)	1 (3.9)	0	0	0
Amp/Ery/Str	7 (6.3)	3(7.2)	4(4.8)	0	4 (6.0)	1 (3.9)	0	2 (14.3)	0
Ery/Nal/Tet	2 (1.8)	0	2(2.9)	0	2 (3.0)	0	0	0	0
Amp/Nal/Str	6 (5.4)	3(7.2)	3(4.4)	0	3 (4.5)	2 (7.7)	0	1 (7.1)	0
Cip/Ery/Str	2 (1.8)	1(2.4)	1(1.5)	0	1 (1.5)	1 (3.9)	0	0	0
Amp/Nal/Str	3 (2.7)	2(4.8)	1(1.5)	1 (50.0)	1 (1.5)	1 (3.9)	0	0	0
Amp/Tet/Str	6 (5.4)	1(2.4)	5(7.3)	0	4 (6.0)	1 (3.9)	1 (100)	0	0
Azm/Ery/Str	2 (1.8)	0	2(2.9)	0	2 (3.0)	0	0	0	0
Amp/Ery/Nal/Str	9 (8.1)	4(9.5)	5(7.3)	0	5 (7.5)	2 (7.7)	0	2 (14.3)	0
Amp/Ery/Chl/Str	4 (3.6)	1(2.4)	3(4.4)	0	3 (4.5)	0	0	1 (7.1)	0
Amp/Nal/Str/Tet	2 (1.8)	0	2(2.9)	0	2 (3.0)	0	0	0	0
Cip/Azm/Chl/Nal	5 (4.5)	3(7.2)	2(2.9)	0	2 (3.0)	2 (7.7)	0	1 (7.1)	0
Amp/Ery/Gen/Str	2 (1.8)	2(4.8)	0	0	0	2 (7.7)	0	0	0
Cip/Amp/Ery/Nal/Tet	3 (2.7)	0	3(4.4)	0	3 (4.5)	0	0	0	0
Amp/Azm/Ery/Nal/Str	4 (3.6)	2(4.8)	2(2.9)	0	1 (1.5)	0	0	2 (14.3)	1 (100)
Amp/Chl/Ery/Nal/Str	1 (0.9)	0	1(1.5)	0	1 (1.5)	0	0	0	0
Cip/Amp/Ery/Nal/Str/Tet	3 (2.7)	1(2.4)	2(2.9)	0	2 (3.0)	1 (3.9)	0	0	0
Cip/Amp/Azm/Ery/Nal/Str	1 (0.9)	1(2.4)	0	0	0	1 (3.9)	0	0	0
Cip/Amp/Azm/Ery/Nal/Str/Tet	1 (0.9)	0	1(1.5)	0	1 (1.5)	0	0	0	0

The antimicrobial resistance was determined using the disk diffusion method. Results are shown as number of isolates with percentage given in parentheses.

a Amp, ampicillin; Azm, azithromycin; Chl, chloramphenicol; Cip, ciprofloxacin; Ery, erythromycin; Gen, gentamicin; Nal, nalidixic acid; Str, streptomycin; Tet, tetracycline.

The analysis of antimicrobial resistance by the broth microdilution method revealed that the majority (94.6%) were resistant to at least one antimicrobial agent, while six (5.4%) isolates were pan-susceptible. The *Campylobacter* isolates displayed resistance most frequently to Amp (75.7%) and less frequently to Cip (7.2%) (Tables 3, 4). In comparison to *C. jejuni*, significantly more (*P* < 0.05) *C. coli* isolates displayed resistance to Str and Amp regardless of the source of the isolates. Seventeen of 42 *C. jejuni* (40.5%) isolates were resistant to three or more antimicrobials, while, 69.6% (48/69) of *C. coli* isolates were resistant to three or more antimicrobials. Approximately 2.4 and 28.6% of *C. jejuni* isolates were resistant to Cip and Gen, respectively (Table 3). Additionally, 10.2% and 2.9% of *C. coli* strains were resistant to Cip and Gen, respectively. While the number of *C. coli* isolates resistant to Gen was significantly different (*P* < 0.01) in comparison to that of *C. jejuni*, there was no significant difference with respect to resistance to Cip. The number of *C. jejuni* (42.9%) isolates resistant to Tyl was not significantly different in comparison to that of *C. coli* (36.2%) (*P*>0.05). However, the number of *C. jejuni* (19.1%) isolates resistant to Ery was significantly different in comparison to that of *C. coli* (66.7%) (*P* < 0.0007).

**Table 3:** **Antimicrobial resistance of *C. jejuni* and *C. coli* isolated from pig, dairy, and beef cattle samples**.

**Resistance profile^a^**	**Resistant isolates**			**Source of isolate and number of resistant isolates (%)**					
				**Pig**		**Dairy cattle**		**Beef cattle**	
	**Number (%)**	***C. jejuni* (*n* = 42)**	***C. coli* (*n* = 69)**	***C. jejuni* (*n* = 2)**	***C. coli* (*n* = 67)**	***C. jejuni* (*n* = 26)**	***C. coli* (*n* = 1)**	***C. jejuni* (*n* = 14)**	***C. coli* (*n* = 1)**
Pan-susceptible	6 (5.4)	6(14.3)	0	0	0	3 (11.5)	0	3 (21.4)	0
Gen	1 (0.9)	1(2.4)	0	0	0	1 (3.9)	0	0	0
Ery	1 (0.9)	1(2.4)	0	0	0	0	0	1 (7.1)	0
Str	2 (1.8)	2(4.8)	0	0	0	0	0	2 (14.3)	0
Gen/Str	2 (1.8)	2(4.8)	0	0	0	2 (7.7)	0	0	0
Gen/Tyl	1 (0.9)	1(2.4)	0	0	0	1 (3.9)	0	0	0
Amp/Tyl	4 (3.6)	4(9.5)	0	0	0	1 (3.9)	0	3 (21.4)	0
Ery/Str	5 (4.5)	2(4.8)	3(4.4)	0	3 (4.8)	1 (3.9)	0	1 (7.1)	0
Amp/Tet	3 (2.7)	1(2.4)	2(2.9)	0	2 (3.0)	0	0	1 (7.1)	0
Str/Tyl	2 (1.8)	1(2.4)	1(1.5)	0	1 (1.5)	1 (3.9)	0	0	0
Cip/Tyl	1 (0.9)	1(2.4)	0	0	0	1 (3.9)	0	0	0
Tet/Str	2 (1.8)	2(4.8)	0	0	0	2 (7.7)	0	0	0
Amp/Str	11 (9.9)	2(4.8)	9(13.0)	2 (100.0)	9 (13.4)	0	0	0	0
Amp/Ery	5 (4.5)	0	5(7.3)	0	5 (7.5)	0	0	0	0
Gen/Amp/Tyl	3 (2.7)	3(7.1)	0	0	0	1 (3.9)	0	2 (14.3)	0
Amp/Str/Tyl	7 (6.3)	3(7.1)	4(5.8)	0	4 (6.0)	2 (7.7)	0	1 (7.1)	0
Ery/Str/Tyl	2 (1.8)	1(2.4)	1(1.5)	0	0	1 (3.9)	0	0	1 (100)
Amp/Ery/Str	15 (13.5)	4(9.5)	11(15.9)	0	11 (16.4)	4 (15.4)	0	0	0
Gen/Amp/Str	2 (1.8)	1(2.4)	1(1.5)	0	1 (1.5)	1 (3.9)	0	0	0
Amp/Tyl/Tet	3 (2.7)	0	3(4.4)	0	3 (4.8)	0	0	0	0
Amp/Str/Tet	2 (1.8)	0	2(2.9)	0	2 (3.0)	0	0	0	0
Gen/Amp/Str/Tyl	3 (2.7)	3(7.1)	0	0	0	3 (11.5)	0	0	0
Amp/Ery/Str/Tyl	12 (10.8)	0	12(17.4)	0	12 (17.9)	0	0	0	0
Cip/Amp/Ery/Str	4 (3.6)	0	4(5.8)	0	4 (6.0)	0	0	0	0
Amp/Ery/Str/Tet	4 (3.6)	0	4(5.8)	0	3 (4.8)	0	1 (100)	0	0
Ery/Str/Tet/Tyl	2 (1.8)	0	2(2.9)	0	2 (3.0)	0	0	0	0
Amp/Ery/Str/Tet/Tyl	1 (0.9)	0	1(1.5)	0	1 (1.5)	0	0	0	0
Cip/Amp/Ery/Str/Tet	3 (92.7)	0	3(4.4)	0	3 (4.8)	0	0	0	0
Gen/Amp/Str/Tet/Tyl	2 (1.8)	1(2.4)	1(1.5)	0	1 (1.5)	1 (3.9)	0	0	0

The antimicrobial resistance was determined using the broth microdilution method. Results are shown as number of isolates with percentage given in parentheses.

a Amp, ampicillin; Cip, ciprofloxacin; Ery, erythromycin; Gen, gentamicin; Str, streptomycin; Tet, tetracycline; Tyl, tylosin.

**Table 4:** **Comparison of antimicrobial resistance of *Campylobacter* spp. identified by disk diffusion and broth microdilution methods**.

**Antimicrobial agent**	**Disk diffusion**				**Broth microdilution**				**Agreement between methods**	
	**No. of isolates**			**% of resistant isolates**	**No. of isolates**			**% of resistant isolates**		
	**S**	**I**	**R**		**S**	**I**	**R**		**Correlation coefficient**	**Kappa values**
**AMINOGLYCOSIDES**										
Gentamicin	65	44	2	1.80%	54	43	14	12.61%	0.0043	0.226
Streptomycin	9	29	73	65.77%	7	21	83	74.77%	0.5671	0.898
**β- LACTAM**										
Ampicillin	13	20	78	70.27%	10	17	84	75.68%	0.7390	0.997
**MACROLIDES**										
Azithromycin	48	48	15	13.51%	–	–	–	–	–	–
Erythromycin	27	38	46	41.44%	28	29	54	48.65%	0.4196	0.695
Tylosin	–	–	–	–	7	61	43	38.74%	–	–
**QUINOLONES**										
Ciprofloxacin	62	33	16	14.41%	78	25	8	7.21%	0.3596	0.739
Nalidixic acid	40	27	44	39.64%	–	–	–	–	–	–
**PHENICOL**										
Chloromphenicol	77	29	5	4.50%	–	–	–	–	–	–
**TETRACYCLINE**										
Tetracycline	48	42	21	18.92%	33	56	22	19.82%	0.8566	0.999

S, susceptible; I, intermediate; R, resistance. MIC and zone of inhibition breakpoints are listed in Kashoma et al. (2015).

Using the broth microdilution and disk diffusion methods, similar results (*P*>0.05) were obtained for five out of six antimicrobial agents (Cip, Str, Amp, Ery, Tet) tested (Table 4). The correlation coefficients between the results obtained from the two methods were 0.0043 for Gen, 0.3596 for Cip, 0.4196 for Ery, 0.5671 for Str, 0.8566 for Tet, and 0.7390 for Amp. Additionally analysis using the Kappa statistics showed that the results obtained using the two tests were mostly in high agreement. The Kappa values were 0.739 for Cip, 0.695 for Ery, 0.898 for Str, 0.977 for Amp, and 0.999 for Tet. Lower agreement was only found for Gen (Kappa = 0.226) (Table 4).

### MLST analysis of *C. jejuni* and *C. coli* isolates

MLST was performed to determine the genetic diversity and clonal origins of the *Campylobacter* isolates that were tested for antimicrobial resistance. A total of 48 different STs were identified for the 111 *Campylobacter* isolates. The *C. jejuni* isolates (*n* = 42) were classified into 26 unique STs of which six (STs 7690, 7697, 7698, 7699, 7700, and 7701) were novel. These novel STs were distributed as follows: three were identified from dairy cattle, two from beef, and one ST from pigs. Nineteen *C. jejuni* STs were assigned to a previously described clonal complex (CC 828), whereas seven STs (including the six new STs) belonged to an undefined clonal complex (Table 5). The *C. coli* isolates (*n* = 69) were classified into 22 STs (Table 5). Two *C. coli* isolates were assigned to a new ST (ST 7683). Fifty two *C. coli* isolates (18 STs) were assigned to a previously described clonal complex (CC 828), while 17 isolates (four STs) belonged to an undefined clonal complex. Overall, these findings imply the occurrence of diverse strains, with majority of STs appearing to not overlap between sources and geographical locations. Only the *C. coli* ST 2713 was identified in two geographical locations (Arusha and Iringa), whereas ST 4309 occurred in pig and dairy samples.

**Table 5:** **Distribution of clonal complexes, sequence types and antimicrobial resistance profiles (Broth microdilution assay) of *C. jejuni* and *C. coli* from pig, dairy, and beef cattle in Tanzania**.

**ST-CC**	**ST**	**No. of isolates**	**Source**	**Geographical location**	**Resistance profiles^a^**
***Campylobacter jejuni***					
828	899	3	Dairy	Morogoro	GenStr, CipTyl, GenTylStr
	1016	1	Dairy	Morogoro	AmpGenTylstr
	1145	3	Dairy	Arusha	AmpEryStr, EryTylStr, AmpGenEryStr
	1201	1	Dairy	Morogoro	Gen
	1465	1	Beef	Arusha	GenTylStr
	1563	4	Dairy	Morogoro	Pan, TylTet, AmpTylStr, AmpEryStr
	1635	2	Dairy	Morogoro	AmpEryTet, AmpEryStr
	1837	2	Beef	Morogoro	Str, EryTet,
	1987	1	Beef	Morogoro	AmpTyl
	2139	1	Beef	Morogoro	Pan
	2702	1	Beef	Morogoro	EryStr
	2878	1	Beef	Iringa	Pan
	4083	1	Dairy	Morogoro	AmpTylStr
	4085	3	Dairy	Morogoro	Pan, StyTet, AmpGenStr
	4679	1	Dairy	Morogoro	AmpGenStr
	7033	1	Beef	Arusha	Str
UC	1240	2	Beef	Morogoro	Pan, TylStrTet
	4609	1	Dairy	Morogoro	GenStr
	7031	1	Beef	Morogoro	AmpTyl
	**7690**	2	Pig	Arusha	AmpStr
	**7697**	2	Dairy	Morogoro	Pan, StrTyl
	**7698**	2	Beef	Arusha	Ery, AmpGenTyl
	**7699**	1	Dairy	Arusha	EryTyl
	**7700**	3	Dairy	Arusha	CipTyl, StrTet, AmpGenTet
	**7701**	1	Beef	Morogoro	AmpTyl
***Campylobacter coli***					
828	872	2	Pig	Iringa	CipEryTylStrTet, EryTylStrTet
	890	3	Pig	Morogoro	AmpEryTylStrTet, CipEryTylStrTet, AmpEryTyrStr
	1056	2	Pig	Morogoro	CipTylStr, AmpEryTylStr
	1117	2	Pig	Iringa	EryStr, AmpEryStrTet
	1153	4	Pig	Morogoro	AmpStr, AmpEryStr, AmpEryStrTyl
	1417	2	Pig	Morogoro	AmpEry, AmpEryStrTyl
	1432	2	Pig	Iringa	AmpStrTyl, AmpTetTyl
	1549	1	Beef	Morogoro	EryStrTyl
	1628	2	Pig	Morogoro	AmpStrTyl, AmpEryStrTyl
	1946	2	Pig	Arusha	AmpTet, AmpStr
	2507	6	Pig	Morogoro	AmpStr, EryStr, StrTyl, AmpEryTyl, AmpEryTetTyl
	2713	4	Pig	Morogoro	AmpStr, AmpTyl, AmpStrTet
	2814	1	Pig	Morogoro	AmpTet
	4085	1	Pig	Morogoro	AmpEryStrTyy
	4309	4	Pig; Dairy	Morogoro	AmpEry, AmpStr, AmpEryStrTet, AmpStrTetTyl
	5250	5	Pig	Morogoro, Arusha	AmpStr, AmpEryTyl, AmpEryStr, AmpEryStrTyl
	5305	2	Pig	Iringa	AmpStrTyl, AmpEryTyl
	5345	7	Pig	Morogoro	EryStr, CipAmp, AmpEryStrTet, AmpEryStrTyl, AmpEryStr, CipAmpEryStr
UC	1469	3	Pig	Arusha	AmpEry, AmpStrTet, AmpEryStrTyl
	1470	4	Pig	Arusha	AmpEryStr, AmpTetTyl,
	6823	8	Pig	Morogoro	AmpEry, AmpEryGen, AmpStrTyl, AmpGenStrTyl, AmpEryStr, AmpEryStrTyl, CipAmpEryStrTet, CipAmpEryStrTet
	**7683**	2	Pig	Iringa	AmpEryStr

UC, undefined clonal complex.

a Amp, Ampicillin; Cip, Ciprofloxacin; Ery, Erythromycin; Gen, Gentamicin; Tet, Tetracycline; Str, Streptomycin; Tyl, tylosin; Pan, Pan-susceptible.

Each antimicrobial resistance profile is underlined.

Bolded numbers show new STs deposited to Pubmlst database.

### Association of sequence types (STs) with antimicrobial resistance pattern

The majority of *C. jejuni* STs (21 STs; 37 isolates) were resistant to multiple antimicrobials, whereas two STs (ST 2139 and ST 2878) were susceptible to all antimicrobials tested by the broth microdilution method (Table 5). Almost all the *C. jejuni* isolates that belonged to undefined CC were resistant to either one or two antimicrobials. In general, *C. coli* isolates showed more diverse antimicrobial resistance profiles in comparison to *C. jejuni* isolates (Table 5). With the exception of ST 2814, which was represented by one isolate that was resistant to two antimicrobial agents, the majority of STs included isolates with different resistance profiles despite belonging to the same ST (Table 5). Mostly*, C. coli* STs were significantly associated with MDR (*P* < 0.05) with the majority (86.7%) of isolates in the undefined complex being resistant to three or more antimicrobials.

## Discussion

We assessed the prevalence, antimicrobial resistance and genetic diversity of *Campylobacter* isolated from pigs, dairy, and beef cattle in Tanzania, where animal husbandry is a very important agricultural activity (MLDF, 2014). In this study, the prevalence of *Campylobacter* in dairy and beef cattle samples was similar to the findings reported in other countries (Gilpin et al., 2008; Ragimbeau et al., 2008; Pradhan et al., 2009; Salihu et al., 2009; Sanad et al., 2011). Furthermore, the overall recovery rate of *Campylobacter* from cattle was significantly different among the three sampling location/regions. While the prevalence of *C. coli* in the pig samples in this study was in agreement with previous reports (Saenz et al., 2000; Jensen et al., 2006), it was also slightly lower than those reported in many other studies (Saenz et al., 2000; Pezzotti et al., 2003; Payot et al., 2004; Mdegela et al., 2011). The differences in *C. jejuni* and/or *C. coli* prevalence within Tanzania and between countries may be due to several factors, including farming and slaughtering practices, geographical locations, or other risk factors, including the concentration of the farms in each location and their proximity to other livestock such as poultry (Moore et al., 2002; Humphrey et al., 2007; Sahin et al., 2015).

Both the agar disk diffusion and the broth microdilution methods have commonly been used to determine antimicrobial resistance in *Campylobacter* (Miflin et al., 2007; Senok et al., 2007). Because of the importance of the implications associated with reporting antimicrobial resistance in human food-borne pathogens, in this study two methods were used in order to generate robust analysis and reduce methodological bias. Our results showed an agreement between these two methods for most of the antimicrobials tested (Table 4), suggesting that either method was generally adequate for analyzing antimicrobial resistance. However, in certain cases such as Gen where the agreement between the two methods was low, multiple susceptibility testing approaches must be adopted and results must be interpreted cautiously to account for methodological variability. The comparison between the two methods is also particularly important for facilitating research conducted in resource limited countries like Tanzania, because it allows the selection of a suitable method for antimicrobial resistance analysis without unnecessary expenditures.

A considerable number of *Campylobacter* isolates in this study was resistant to macrolides (Ery, Tyl, and Azm) (Tables 2, 3). Macrolides such as Tyl are extensively used in Tanzania as therapeutic agents for treatment of cattle respiratory conditions (Kashoma et al., 2015). The use of Tyl in animals for the purpose of either treatment or growth promotion contributes to the selection of resistant *Campylobacter* strains to macrolides including Ery (Juntunen et al., 2010). These observations suggest a possible association between the heavy use of Tyl in Tanzania and the increase in the resistance *Campylobacter* to Tyl and Ery observed in this study. Furthermore, the high resistance to macrolides (Ery) in *Campylobacter* isolated from humans in Tanzania (Komba et al., 2015) highlights the need for understanding the impact of the use of antimicrobials in animal agriculture on the rise of resistant pathogens in food animals and humans. This further emphasizes the need for *Campylobacter* surveillance and control studies in Tanzania.

In this study, a relatively high level of resistance to Nal (47.6% *C. jejuni* and 34.8% *C. coli*) was observed, while the number of Cip resistant *Campylobacter* isolates (11.7% and 7.2% using the disk diffusion and broth microdilution methods, respectively) was relatively low (Tables 2, 4). The wide-spread resistance to Nal corroborated reports on *Campylobacter* from different food animals/products in other countries (Taremi et al., 2006; Bostan et al., 2009; Dabiri et al., 2014), while Cip resistance was generally lower than elsewhere (Taremi et al., 2006; Wieczorek and Osek, 2013). Furthermore, in a previous study on beef carcasses and raw milk in Tanzania (Kashoma et al., 2015), it was observed that *C. jejuni* (65.7%) and *C. coli* (63.2%) isolates were resistant to Nal, while 9.3–11.8% were resistant to Cip. The resistance to Cip in isolates originating from food animals/products in Tanzania is of concern, because this antimicrobial is used for treatment of human campylobacteriosis. Currently, the factors that are promoting Cip resistance in Tanzanian food-associated isolates are not clear, especially because it is known that this antimicrobial is not commonly used in animal agriculture in Tanzania. However, enrofloxacin is licensed for therapeutic use in poultry against colibacilliosis, pasteurellosis, and mycoplasmosis (Mubito et al., 2014), which may result in the selection of resistance of *Campylobacter* to fluoroquinolones. These resistant isolates might be transmitted to other food animals that are in the proximity of poultry via a variety of vehicles, including common farm workers and/or flies or other critters (McDermott et al., 2002; Van Boven et al., 2003; Stapleton et al., 2010). Furthermore, it is possible that Cip resistant isolates might have originated from humans where this antimicrobial is commonly used. This might be supported by a recent report showing that a relatively high percentage (22.1%) of human *Campylobacter* isolates were resistant to Cip in Tanzania (Komba et al., 2015). Regardless, the determination of factors that select for Cip resistant isolates will require further investigation.

In this study, a range of resistance (low to high number of isolates) was observed for different antimicrobials. Specifically, a relatively low number of isolates were resistant to Gen (1.8–12.6% of isolates depending on the testing method) and Chl (6.3%), respectively. Generally, Chl and Gen resistance in *Campylobacter* is known to be low (Fallon et al., 2003; Kassa et al., 2007). Furthermore, a previous study in Tanzania showed that 13% and 11.8% of the *Campylobacter* isolated from beef carcasses and raw milk were resistant to Chl and Gen, respectively (Kashoma et al., 2015). Since Tets are widely used in humans and as therapeutics or feed additives in livestock and poultry in Tanzania, a high number of Tet resistant isolates was expected. For example, Komba et al. (2015) reported 76.5% and 57.1% of human-associated *C. jejuni* and *C. coli* were resistant to Tet in Tanzania. However, a moderate resistance to Tet (19.8% of the isolates) was observed in this study. The reasons behind this are currently not clear and require further analysis. In contrast, *Campylobacter* can inherently display resistance to β-lactams (including Amp) (Engberg et al., 2006; Li et al., 2007). Consequently, the high resistance to Amp observed in this study was not surprising.

In recent years multidrug resistant *Campylobacter* strains have been increasingly reported worldwide, which is now recognized as a major emerging public health concern. The numbers of multidrug resistant isolates (40% *C. jejuni* and 69.9% *C. coli*) are comparable to those reported in other countries (Van Looveren et al., 2001; Pezzotti et al., 2003; Wieczorek and Osek, 2013). Furthermore, analysis of human-associated *Campylobacter* in Tanzania showed that 77.9% of the isolates were resistant to more than six of the tested antimicrobials, while 19.9% were resistant to all tested antimicrobials (*n* = 12) (Komba et al., 2015). While the contribution of food animal-associated isolates to the MDR in human isolates is currently unknown, this is a point of serious concern that suggests that Tanzania, like other countries, has to devise stringent control and regulatory measures to reduce MDR isolates in the food chain.

MLST analysis classified the tested isolates into 48 STs. Almost 71.2% of the isolates were assigned to known CC, while 28.8% could not be traced to known lineages. These findings highlight the diversity of *Campylobacter* genotypes and suggest that certain food-animal associated isolates might have evolved and adapted to Tanzanian farm animals and/or farming practices. In this study, the majority of *C. coli* and *C. jejuni* isolates belonged to ST 828 clonal complex, which is associated mainly with isolates from agricultural and environmental sources and human clinical cases (Sheppard et al., 2010). Also, researchers have reported the presence of progenitor strains of the ST 828 complex in human, swine, poultry, and cattle from different parts of the world (Dingle et al., 2005; Miller et al., 2006; Sanad et al., 2011; Kashoma et al., 2014). This is important, because the contributions of various possible sources of infection, including food animals, to the burden of human campylobacteriosis in Tanzania is not clearly defined and require further investigations.

## Conclusions

In the present study, a high prevalence and genotypic diversity of *Campylobacter* from pigs, dairy, and beef cattle in Tanzania was observed. The majority of the *Campylobacter* isolates examined were resistant to multiple antimicrobials, which was confirmed using two different methods Therefore, prudent use of antimicrobials in veterinary/farming practices remains essential to reduce the pool of antimicrobial resistant pathogens that might impact human health. Collectively, this study highlights the need for continuous efforts to control *Campylobacter* colonization in farm animals in order to limit the transmission of these pathogens to humans.

## Author contributions

GR, RK, and IPK conceived the study. IPK, IIK, AK, and BK performed the experiments. IPK, IIK, WG, RK and GR analyzed the data. IPK, IIK, and GR wrote the manuscript. All authors read and approved the final manuscript.

### Conflict of interest statement

The authors declare that the research was conducted in the absence of any commercial or financial relationships that could be construed as a potential conflict of interest.
